# Prosthetic Management of Congenital Palatal Defect in a Neonate: A Case Report on Obturator Efficacy

**DOI:** 10.7759/cureus.63708

**Published:** 2024-07-02

**Authors:** Swamini S Gabhane, Meenal S Pande, Nilima R Thosar, Ramakrishna Yeluri, Monika Khubchandani, Neha Pankey

**Affiliations:** 1 Pediatric and Preventive Dentistry, Datta Meghe Institute of Higher Education and Research, Wardha, IND

**Keywords:** nasal regurgitation, obturator, pediatric dentistry, feeding device, cleft palate

## Abstract

Neonates with cleft palate exhibit a malformed maxillary arch since birth. Newborns with various types of clefts exhibit multiple issues, primarily associated with their feeding habits. Feeding these children is crucial, as evidence indicates that newborns with this congenital deformity exhibit a slower growth rate compared to those without this condition. To mitigate these challenges, the conventional line of treatment for these children is obturator therapy to facilitate sucking or feeding followed by various surgical procedures. The following case report describes a 2-day-old girl, who reported with her parents to the Department of Pediatric Dentistry, to seek treatment for congenital cleft present in her palate as it was interfering with her feeding habits. A feeding appliance was made with a direct technique to help the parents improve feeding habits. It also regulates milk flow by sealing the area separating the oral and nasal cavities. This feeding appliance is placed over the child's hard palate, creating a contact point that facilitates milk expression from the mother’s mammary gland and making it easier for the neonate to compress the nipple. It shortens the time needed for feeding, eases feeding, and lessens nasal regurgitation.

## Introduction

The term "cleft palate," also known as "palatochisis," refers to a type of congenital deformity in which aberrant facial development occurs during gestation as a result of complicated genetic and environmental variables [1]. This condition can also occur simultaneously as a combined cleft lip and palate deformity. Clefts of the lip and palate are the most common congenital craniofacial malformations in children in India with prevalence ranging somewhere between 27000 and 33000 clefts per year, which in turn cause decreased nutrition, thus reducing weight [2]. Infants with orofacial clefts have trouble sucking at birth. It is the primary role of not only the pedodontist but also the orthodontist, who can build an obturator since it allows for improved feeding, which encourages weight gain and is a requirement for surgically addressing the abnormalities. It also serves as a temporary aid for speech articulation until a permanent surgical repair can be accomplished [3]. The function of the palatal obturator is significant in minimizing the duration of the feeding session, supplementing milk consumption, diminishing nasal cavity and respiratory tract infections, and lowering the frequency of otitis media, a pragmatic strategy, motivated by a deeper understanding of the issue and simultaneous actions to fix the feeding problems, would provide impressive outcomes promptly [4]. Numerous organizations have already started significant efforts to lessen the burden as a result of the shift in the way cleft care is delivered globally, all to sum up that the goal of obturator therapy is to partially restore the congenital palatal deformity [5]. Feeding obturators can assist a baby in developing suction and make feeding easier [6]. The purpose of this case study is to demonstrate the clinical considerations, fabrication process, and functional results involved in the appropriate use of an obturator in a patient with cleft palate.

## Case presentation

**Figure 1:**
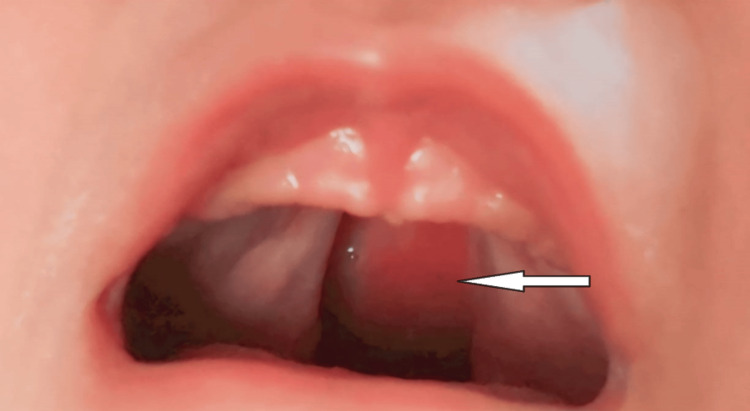
Unilateral cleft involving the hard palate.

A 2-day-old female child was brought by her parents to the Department of Pediatric and Preventive Dentistry, Sharad Pawar Dental College, Wardha with a chief complaint of frequent nasal regurgitation of milk due to a congenital defect present in her palate. The child struggled to latch and nurse effectively, leading to concerns about inadequate nutrition during feeding attempts. The child weighed about three pounds, and history-taking revealed that the mother had a consanguineous marriage, and the child had a normal delivery. The extraoral examination revealed a grossly symmetrical face, with normal lips, and no visible nasal deformities. There were no anomalies or lesions on the skin of the face. The infant's lower jaw examination revealed normal size and position, the facial musculature revealed adequate muscle tone, and there were no signs of facial nerve dysfunction. A unilateral cleft involving the hard palate was found during the intraoral examination; this was in accordance with Veau's Classification of Cleft Lip and Palate (Figure 1).

**Figure 2:**
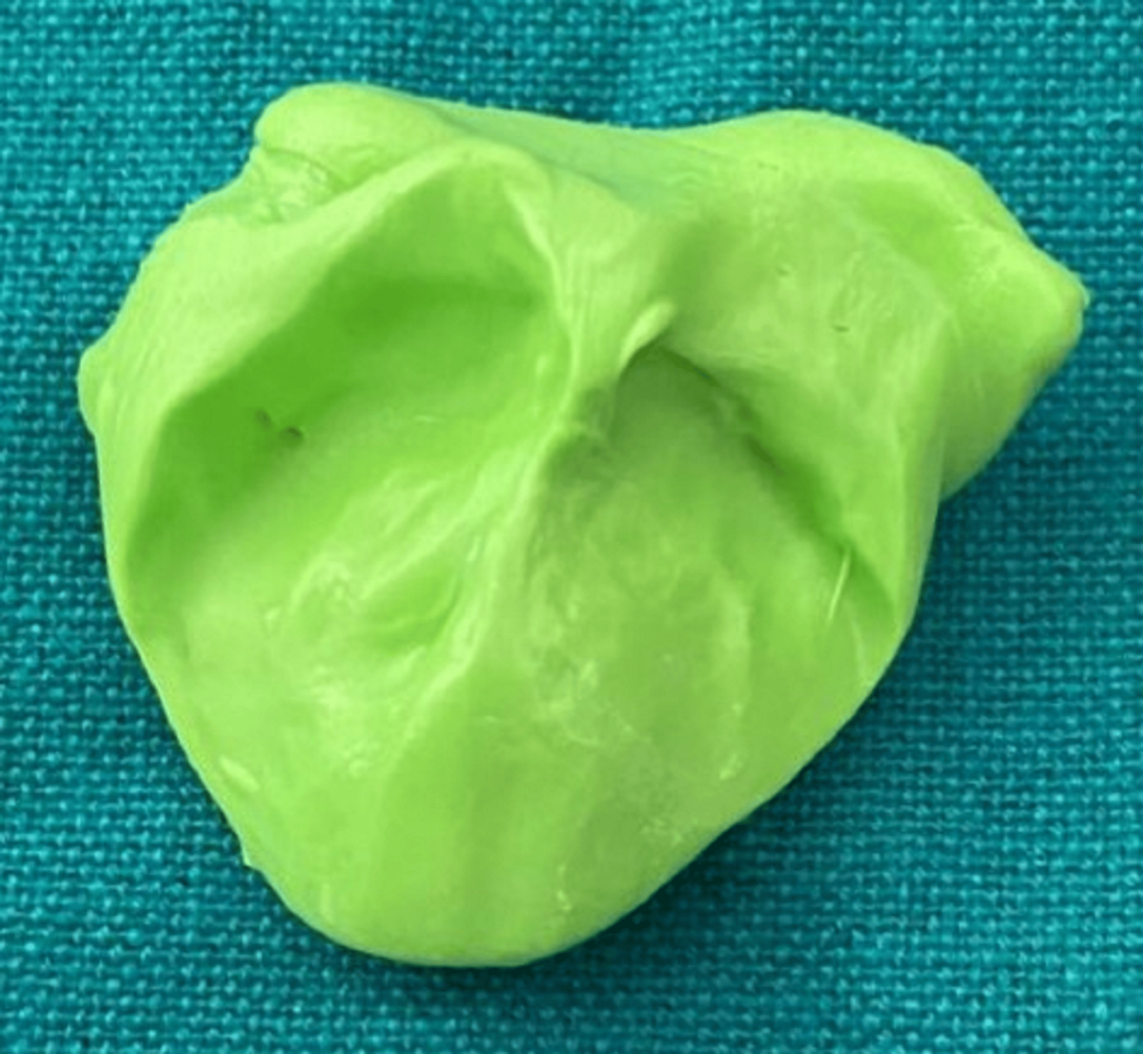
Impression using a-silicon impression material (putty fast set).

Written consent was obtained from the parents for the initiation of obturator therapy. The parents were educated about the condition their daughter was suffering from and were further explained about the treatment options and further outcomes that mainly alter the quality of life. The first step involved in obturator fabrication is impression making in which the position of the infant was upright. It should be mentioned that the infant should cry while impression-making. Then selection of a suitable impression material that is non-toxic, tasteless, and sets quickly was made. So, a silicon impression material (putty fast set) was used to create an impression (Figure 2).

**Figure 3:**
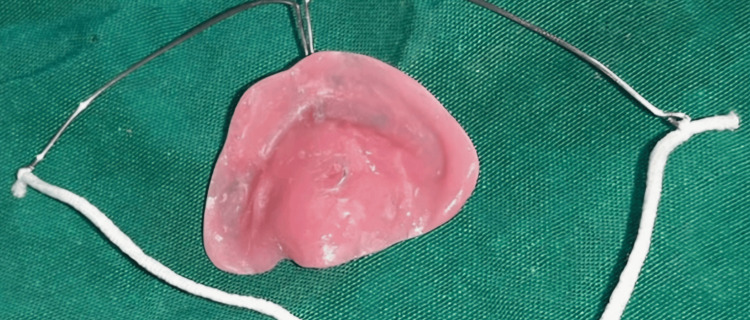
The fabricated obturator for the patient.

It was taken care that no aspiration of any excess material happened. This was mainly made by the upright position of the patient. Excess material was cut using a sharp instrument, then the cast was poured with dental stone, and modeling wax was used to block out the area of the cleft. The obturator was then made using a sprinkle-on technique with cold-cure acrylic manufactured by Dental Products of India (Mumbai, India). After appropriate trimming using Acrylic Trimming Bur T.C Bur (F06), the fabricated obturator was extra-orally examined for any rough surface and then placed and checked intraorally to see if it initiated a sucking reflex (Figure 3).

**Figure 4:**
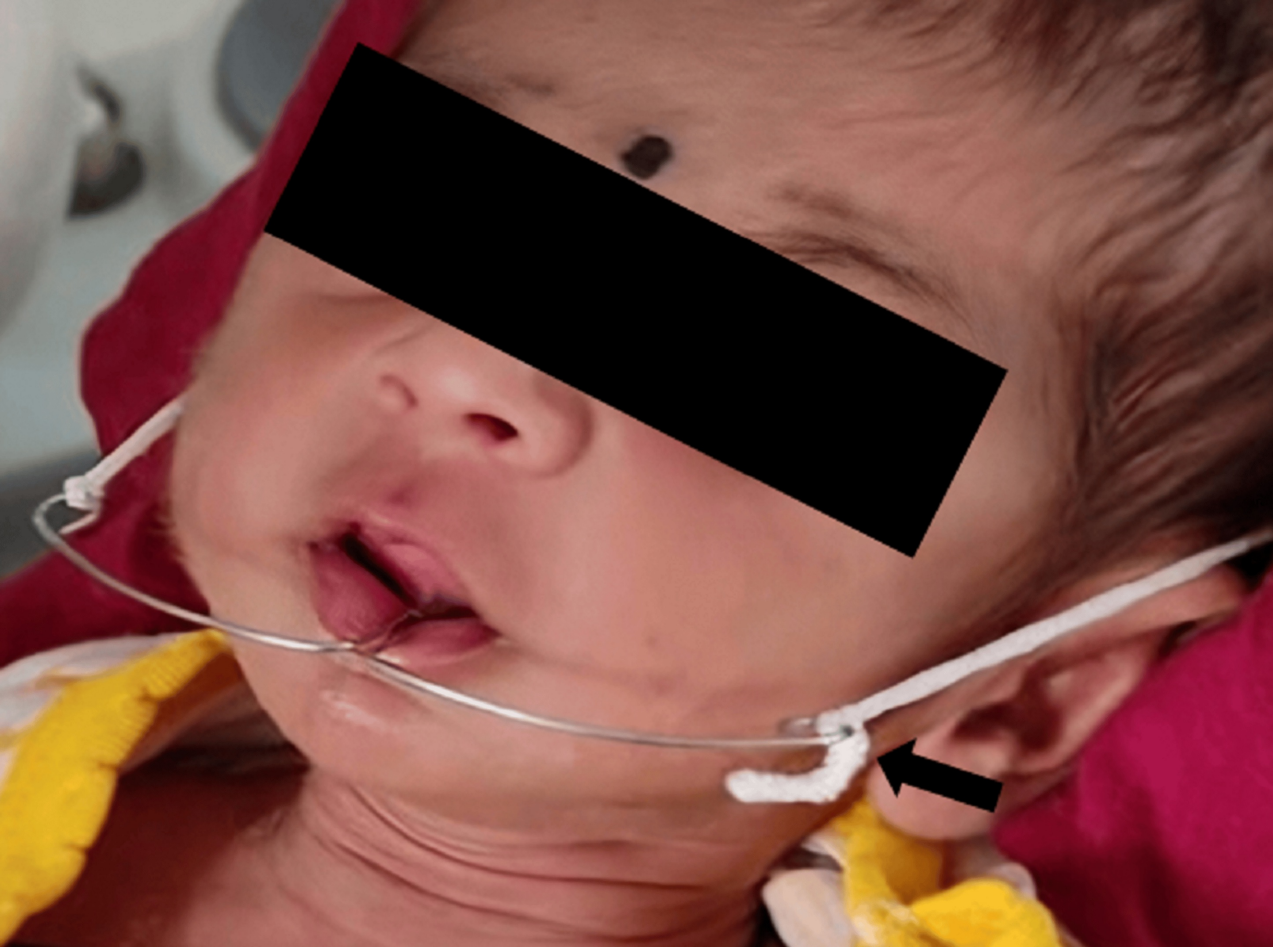
Post-operative image of intraoral application of obturator.

Instructions were given to the mother regarding the storage and use of the obturator, like it should be kept in a clean and dry place when not in use, regularly inspecting the surface for any signs of wear or tear, and after feeding, the complete infant oral cavity and obturator should be cleaned with a soft cloth soaked in warm water. The mother was also educated properly regarding the purpose of the feeding plate during its insertion. In the Department, the fabricated obturator was tried in the infant's mouth, and the mother was asked to feed the baby - no nasal regurgitation of milk was noticed (Figure 4). Following a 24-hour period, the infant was evaluated again and further appointments were planned to promote normal growth of the jaw.

## Discussion

Around one in every 700 babies is born with one of the common birth abnormalities - cleft lip or cleft palate [6]. There is obvious concern about feeding these children, and there is evidence that children with clefts grow more slowly than their non-cleft counterparts [7]. A plastic surgeon, otolaryngologist, prosthodontist, pedodontist, orthodontist, speech therapist, audiologist, and social worker make up the team for the surgical management of a child [8]. To treat such patients, there is a treatment protocol as shown in Table 1. Typically, a child with a cleft lip is fixed according to Millard’s rule of 10 - the neonate should be 10 weeks of age, weigh 10 pounds, and have hemoglobin exceeding 10 g/dl before undergoing surgery [9].

**Table 1:** The treatment protocol for treating cleft lip and palate patients.

CONDITION	TREATMENT
1. Before Birth	Diagnosis and Consultation [10]
2. 1-3 months	Obturator [Feeding plate], Nasoalveolar molding [10]
3. 3-4 months	Lip surgery [10]
4. 1 year	Palatoplasty [10]
5. 2.5 -5 years	Velopharyngeal insufficiency therapy, Speech therapy [10]
6. 6-9 years	Alveolar bone grafting [10]
7. 16-18 years	Orthognathic surgery [10]

Among all these treatment approaches, the first-line treatment option for cleft patients is the fabrication of an obturator [11]. Neonates whose only medical condition is a cleft palate, or both cleft lip and cleft palate, swallow normally but suck abnormally because of faulty muscle attachments and communication between the nose and the oral cavity. The elevator and tensor muscles that attach along the back of the hard palate and extend along the midline in normal situation fail to do so when a cleft is present. It is practically hard to isolate the oral cavity, generate suction, and establish negative pressure because of this aberrant design and anatomy. As a result, babies that have orofacial clefts swallow normally but suck abnormally [12]. The feeding appliance fulfills this need. It is a passive prosthesis device intended to restore the natural contours of the hard palate and cleft alveolus. The difficulties in fabricating this appliance in a baby include the oral cavity's limitations, the cleft's variability in size, position, and severity, and the infant's resistance to cooperation. A crucial step is impression. To prevent inadvertent swallowing of the impression material, a number of positions have been used, such as, face down, upright, and even upside down [13].

The obturator facilitates nursing, inhibits nasal regurgitation, promotes oral and facial development, strengthens the palatal shelves, lessens ear infections, and stops tongue distortion and nasal septum irritation. It also leads to normal growth and expansion of maxillary arch [14]. All of these benefits not only help the infants but also provide psychological support to parents. In such situations, the contribution of pediatric dentist is invaluable. In this case, the obturator or the feeding plate not only helped the child get necessary nutrition but also to get ready for the future surgical approaches that are needed to be planned as and when required [15].

## Conclusions

In this case report, we examined the use of an intraoral obturator for an infant with a palatal defect. The obturator will support the child until surgery can be done as a temporary measure. In the pre- and post-operative stages, the use of an intraoral obturator can be quite important as it provides immediate functional benefits and improves the overall course of treatment. To further enhance patient comfort and efficacy, future recommendations should focus on advancing obturator design and conducting further studies on the long-term advantages. In general, the intraoral obturator continues to be an important tool for treating individuals with cleft palate, greatly enhancing their quality of life and rehabilitation.

## References

[1] Farronato G, Cannalire P, Martinelli G, Tubertini I, Giannini L, Galbiati G, Maspero C (2014). Cleft lip and/or palate: review. Minerva Stomatol.

[2] Mossey P, Little J (2009). Addressing the challenges of cleft lip and palate research in India. Indian J Plast Surg.

[3] Agarwal A, Rana V, Shafi S (2010). A feeding appliance for a newborn baby with cleft lip and palate. Natl J Maxillofac Surg.

[4] RN Alirhayim, AJ Mohammed (2022). Feeding obturator: indications, types, and benefits. a literature review. Azerbaijan Med Assoc J.

[5] Chen J, Yang R, Shi B, Xu Y, Huang H (2022). Obturator manufacturing for oronasal fistula after cleft palate repair: a review from handicraft to the application of digital techniques. J Funct Biomater.

[6] Salari N, Darvishi N, Heydari M, Bokaee S, Darvishi F, Mohammadi M (2022). Global prevalence of cleft palate, cleft lip and cleft palate and lip: a comprehensive systematic review and meta-analysis. J Stomatol Oral Maxillofac Surg.

[7] Bowers EJ, Mayro RF, Whitaker LA, Pasquariello PS, LaRossa D, Randall P (1987). General body growth in children with clefts of the lip, palate, and craniofacial structure. Scand J Plast Reconstr Surg Hand Surg.

[8] Hartzell LD, Kilpatrick LA (2014). Diagnosis and management of patients with clefts: a comprehensive and interdisciplinary approach. Otolaryngol Clin North Am.

[9] Schalet G, Langlie J, Kim M, Thaller S (2023). The rule of 10s for cleft repair: a historical review of the literature. J Craniofac Surg.

[10] Nahai FR, Williams JK, Burstein FD, Martin J, Thomas J (2005). The management of cleft lip and palate: pathways for treatment and longitudinal assessment. Semin Plast Surg.

[11] Naveen BH, Prasad RS, Kashinath KR, Kumar S, Kalavathi SD, Laishram N (2019). An innovative modified feeding appliance for an infant with cleft lip and cleft palate: a case report. J Family Med Prim Care.

[12] Devi ES, Sai Sankar AJ, Manoj Kumar MG, Sujatha B (2012). Maiden morsel - feeding in cleft lip and palate infants. J Int Soc Prev Community Dent.

[13] Ravichandra KS, Vijayaprasad KE, Vasa AA, Suzan S (2010). A new technique of impression making for an obturator in cleft lip and palate patient. J Indian Soc Pedod Prev Dent.

[14] María CA, María MC, Emilia CG (2022). Maternal perception of breastfeeding in children with unilateral cleft lip and palate: a qualitative interpretative analysis. Int Breastfeed J.

[15] Campbell A, Costello BJ, Ruiz RL (2010). Cleft lip and palate surgery: an update of clinical outcomes for primary repair. Oral Maxillofac Surg Clin North Am.

